# Carotid blood flow changes following a simulated end-inspiratory occlusion maneuver measured by ultrasound can predict hypotension after the induction of general anesthesia: an observational study

**DOI:** 10.1186/s12871-023-02393-6

**Published:** 2024-01-03

**Authors:** Guangshan Jin, Fuqiang Liu, Yiwen Yang, Jiahui Chen, Qian Wen, Yudong Wang, Ling Yu, Jianhua He

**Affiliations:** 1 School of Anesthesiology, Xuzhou Medical University, Jiangsu, China; 2 Department of Anesthesiology, Jiangsu Cancer Hospital, The Affricated Cancer Hospital of Nanjing Medical University, Jiangsu, China; 3 Department of Ultrasound, Affiliated Hospital of Integrated Traditional Chinese and Western Medicine, Nanjing University of Chinese Medicine, Jiangsu, China

**Keywords:** Simulated end-inspiratory occlusion test, Ultrasonography, Corrected flow time, Peak blood flow velocity, Post-induction hypotension

## Abstract

**Background:**

The primary purpose of this study was to investigate the predictive value of alterations in cervical artery hemodynamic parameters induced by a simulated end-inspiratory occlusion test (sEIOT) measured by ultrasound for predicting postinduction hypotension (PIH) during general anesthesia.

**Methods:**

Patients undergoing gastrointestinal tumor resection under general anesthesia were selected for this study. Ultrasound has been utilized to assess hemodynamic parameters in carotid artery blood flow before induction, specifically focusing on variations in corrected flow time (ΔFTc) and peak blood flow velocity (ΔCDPV), both before and after sEIOT. Anesthesia was induced by midazolam, sufentanil, propofol, and rocuronium, and blood pressure (BP) and heart rate (HR) were recorded within the first 10 min following endotracheal intubation. PIH was defined as fall in systolic blood pressure (SBP) or mean arterial pressure (MAP) by > 30% of baseline or MAP to < 60 mm Hg.

**Results:**

The area under the receiver operating characteristic curves (AUC) for carotid artery ΔFTc was 0.88 (95%CI, 0.81 to 0.96; *P* < 0.001), and the optimal cutoff value was -16.57%, with a sensitivity of 91.4% and specificity of 77.60%. The gray zone for carotid artery ΔFTc was -16.34% to -15.36% and included 14% of the patients. The AUC for ΔCDPV was 0.54, with an optimal cutoff value of -1.47%. The sensitivity and specificity were calculated as 55.20% and 57.10%, respectively.

**Conclusion:**

The corrected blood flow time changes in the carotid artery induced by sEIOT can predict hypotension following general anesthesia-induced hypotension, wherein ΔFTc less than 16.57% is the threshold.

**Trial registration:**

Chinese Clinical Trial Registry (www.chictr.org.cn; 20/06/2023; ChiCTR2300072632).

The occurrence of postinduction hypotension (PIH) is a frequently observed adverse reaction during the administration of general anesthesia. Following the induction of general anesthesia, patients face a significant risk of experiencing PIH due to the cardiovascular depressant and vasodilator effects induced by anesthetic agents [1]. Anesthesiologists frequently overlook PIH due to factors such as patient positioning, adjustment of ventilator parameters, and post-tracheal intubation documentation of anesthesia orders. PIH below the lower limit of vascular autoregulation curve may lead to ischemia in vital organs such as the heart, brain, and kidneys, closely correlating with perioperative myocardial injury, acute kidney injury, and stroke [2–5]. Consequently, PIH contributes to increased postoperative mortality rates and prolonged hospital stays [6, 7].

Currently, there is yet to be universally accepted definition of PIH. The duration and severity of hypotension, along with the administration of vasoactive medications, serve as the criteria for defining PIH. The predictors of PIH include age, preinduction systolic blood pressure (SBP) and mean arterial pressure (MAP), American Society of Anesthesiologists (ASA) class III and IV, doses of propofol and fentanyl, and preoperative medication [8–10]. Additionally, patients undergoing elective surgery may experience hypovolemia due to various factors, such as preoperative fasting and bowel preparation, which increases the risk of hypotension. Recently, MIYAZAKI [11] and Shao [12] et al. demonstrated that resting-state pupil diameter and pupil contraction velocity have been identified as reliable predictors of PIH. However, it is essential to note that some anesthetics can affect pupil size and other factors such as age and blink rate. Therefore, further investigation is necessary to identify more robust predictors in clinical practice.

In recent years, ultrasound technology has been significantly utilized during perioperative period, particularly in assessing surgical patient volume. Zhang et al. [13] showed that preanesthetic measurement of the inferior vena cava collapse index (IVC-CI) using ultrasound can effectively predict hypotension after induction. However, the measurement of IVC diameter using ultrasound is influenced by respiratory variations, and the patient transitioning from spontaneous breathing to positive pressure ventilation postinduction. As a result, certain limitations are imposed on the clinical applicability of this index. Several studies have investigated carotid indicators, including systolic flow time (the duration of the heart cycle in contraction), carotid intima-media thickness, carotid blood flow velocity, and carotid blood flow [5, 14–17]. Corrected flow time (FTc) and peak velocity variation (CDPV) have been utilized to identify fluid responsiveness in diverse clinical scenarios [18]. Due to its superficial location, ease of measurement, and minimal influence from respiration, the hemodynamic parameters in the carotid artery can effectively reflect volume responsiveness during spontaneous breathing and mechanical ventilation [19].

In a pilot study [20], flow measures of the common carotid artery reflected transient changes in stroke volume and velocity time integral (VTI) in the descending aorta during simulated end-inspiratory/end-expiratory occlusion tests(sEIOT/sEEOT) conducted on healthy volunteers. Aya et al. [21] found that alterations in FTc resulting from recruitment maneuvers performed by anesthesiologists during surgery can serve as indicators for assessing intraoperative patient fluid responsiveness. However, no previous reports have investigated whether carotid Doppler ultrasound combined with sEIOT can predict PIH in spontaneously breathing patients. Therefore, this study aims to explore the predictive value of sEIOT-induced changes in carotid corrected blood flow time and peak blood flow velocity based on ultrasound measurements of carotid blood flow parameters before anesthesia induction.

## Materials and methods

### Study design and participants

The Ethics Committee of Jiangsu Cancer Hospital has approved this prospective observational study (2023Ke-Kuai029), registered in the Chinese Clinical Trial Registry (www.Chictr.org.cn; ChiCTR2300072632). Written informed consent was obtained from the patients and their families. From June to September 2023, eligible participants included individuals aged 18–64 years who underwent gastrointestinal tumor resection, had an ASA II-III, and could cooperate with sEIOT before surgery. Exclusion criteria included SBP > 160 mmHg and other significant cardiovascular and cerebrovascular diseases; a history of atherosclerosis; severe hypertension; nonsinus rhythm; heart valve disease; cardiac insufficiency (left ventricular ejection fraction < 50%, right ventricular dysfunction); severe pulmonary disease, respiratory insufficiency or hemodynamic instability; oral intake of ACEI or ARB drugs before surgery; patients with A-V fistula and dialyzed patients. Participants failing to comply with sEIOT during the trial or those without clear and effective images and measurement data were also excluded. Difficult tracheal intubation was defined as requiring three or more attempts for intubation or an intubation time exceeding 1 min. Additionally, baseline MAP < 70 mmHg or SBP < 90 mmHg served as exclusion criteria.

### Carotid artery ultrasonography

Following established protocols, the ultrasound examination was conducted using a ultrasound machine (Mindray, Te 7, China). The patient assumed a supine position, ensuring full exposure of the left side of the neck. A linear array probe (7–12 MHz) was employed to precisely identify the transverse section of the common carotid artery below the thyroid cartilage. The probe was placed 2 cm below the bifurcation of the common carotid artery, three consecutive stable cardiac cycles were obtained, and an acceptable level of image quality was achieved. Carotid Flow time (FT), defined as the time interval between the systolic rise phase and dicrotic notch, was measured using the pulsed Doppler technique and corrected for heart rate according to Wodey’s formula: FTc = FT + [1.29 × (HR-60)]. Subsequently, FTc could be calculated from this equation. Carotid Doppler peak velocity (CDPV) was assessed before and after sEIOT within one respiratory cycle. The ultrasound measurements were conducted independently by a highly skilled investigator with extensive ultrasound manipulation experience (L, Y).

### Trial intervention and outcomes

Patients were transported to the preoperative waiting area, and heart rate (HR), transcutaneous oxygen saturation (SPO_2_), SBP, diastolic blood pressure (DBP), and MAP were monitored. Before measurement, patients were instructed to maintain quiet breathing for 15 s, followed by a deep breath-hold maneuver. Then, the patient was instructed to close his mouth and hold his breath for at least 30 s while connecting the pressure measurement device to ensure a constant pressure of 25–30 cmH_2_O in the mouth [20].

**Fig. 1:**
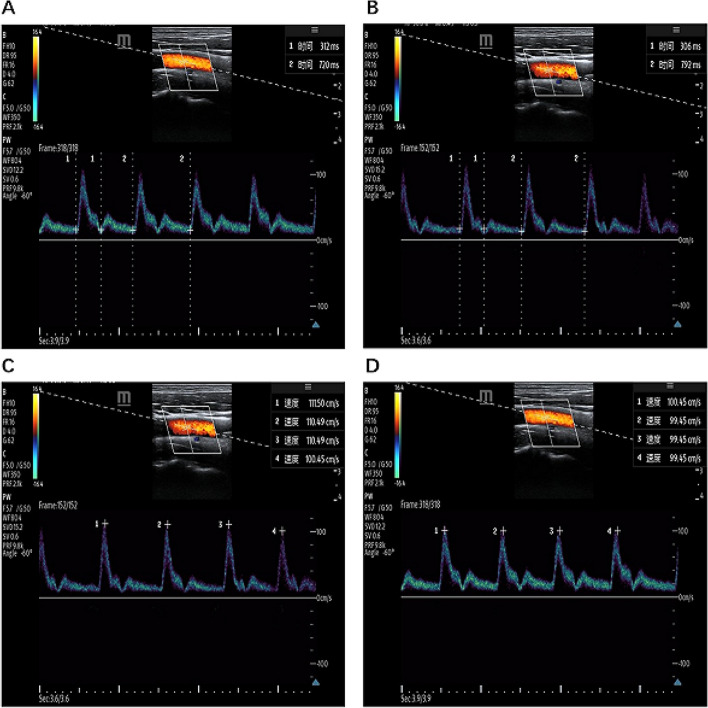
**A** Time1 is the FT before sEIOT, which is corrected by heart rate to obtain FTc, that is, FTc pre in the formula (FTc post − FTc pre)/ FTc pre × 100; **B** Time1 is the FT after sEIOT, which is corrected by heart rate to obtain FTc, that is, FTc post in the formula (FTc post − FTc pre)/ FTc pre × 100; **C** Velocity1 is the CDPVpre in the formula (CDPVpost − CDPVpre)/ CDPVpre × 100,which measurements occurred before the sEIOT; **D** Velocity1 is the CDPVpost in the formula (CDPVpost − CDPVpre)/ CDPVpre × 100, which measurements occurred after the sEIOT

The carotid artery FT and CDPV were measured before and after achieving the prescribed breath-hold time and pressure. The ΔFTc was calculated using the formula (FTc post − FTc pre)/ FTc pre × 100. Similarly, the ΔCDPV was obtained by calculating (CDPVpost − CDPVpre)/ CDPVpre × 100. Consider three trials to determine the average values to decrease measurement error [21, 22] (Fig. 1).

### Anesthesia protocol

No medication was administered to the patients before anesthesia, and adherence to routine fasting protocols was mandatory. In the operating room, continuous monitoring was performed for ECG, SPO2, SBP, DBP, MAP, and BIS. Intravenous access was established to administer sodium acetate Ringer’s solution at a rate of 10 ml/kg/h while simultaneously providing mask oxygen inhalation. Anesthesia induction followed a standardized regimen consisting of midazolam (0.1–0.2 mg/kg), sufentanil (0.4–0.5 μg/kg), propofol (1.0–2.0 mg /kg), and rocuronium (0.6–0.9 mg/kg). Tracheal intubation was performed using video laryngoscopy 2 min after anesthesia induction commenced. Patients were pressure-controlled volume guaranteed(PCV-VG) ventilated (tidal volume-VT: 6–8 ml/ kg; respiratory rate-RR: 10–12 breath/min) to maintain an end-tidal carbon dioxide partial pressure between 35 and 45 mmHg. The PIH utilized in this study, which was defined as a reduction of more than 30% from the baseline value in SBP and MAP within 10 min following endotracheal intubation, or when MAP < 60mmHg [4, 23]. Noninvasive cuff measurements of SBP, DBP, MAP, and HR were recorded at 3, 5, 7, and 9 min after tracheal intubation. Following intubation, a strict protocol should be followed during the initial 10-min period to minimize stimulation and maintain the patient’s position without any changes. Severe (MAP less than 60 mmHg) or prolonged (duration greater than or equal to 2 min) episodes of hypotension were treated using intravenous boluses of ephedrine (3 mg) or phenylephrine (100 μg). Atropine (0.3 mg) was used for significant bradycardia (HR less than 40 beats/min).

### Statistical analysis

PASS 15.0 software was used to calculate the sample size. Wang et al. [24] previously reported an area under the receiver operating characteristic curve (AUC) of 0.87 for FTc in predicting hypotension after induction of general anesthesia in elderly patients. In this study, we assumed a conservative predictive power for ΔFTc and set the AUC to 0.70 accordingly. Based on these assumptions, a needed sample size of 92 patients was calculated with α = 0.05 and 1-β = 0.80, considering a dropout rate of 10%. Therefore, a total of 102 patients were recruited for analysis.

Statistical analysis was conducted using SPSS27.0 software, with categorical data presented as the number of cases (percentage) and compared between groups using the χ2 test. Continuous data with a normal distribution were expressed as mean ± standard deviation and analyzed between groups using an independent sample t-test or within groups using a paired sample t test accordingly. Nonparametric continuous data were represented as median (lower quartile - upper quartile) [M (P25, P75)] and compared using rank-sum test. A significance level of *P* < 0.05 was considered statistically significant.

Based on the trial findings, we constructed a receiver operating characteristic (ROC) curve to illustrate the performance characteristics of the subjects. Additionally, we calculated the area under the curve (AUC) and conducted statistical analysis to evaluate the sensitivity and specificity of ΔFTc and ΔCDP in predicting hypotension after anesthesia induction. Furthermore, the best cutoff value was chosen to maximize the Youden index. The gray area method represented the time change values for carotid artery corrected blood flow. The pearson correlation test was used to analyze the correlation between sEIOT-induced ΔFTc and the percentage decrease in hemodynamic indexes after anesthesia induction compared to baseline. Multivariate logistic regression analysis was performed to identify influencing factors associated with hypotension after anesthesia induction.

## Results

**Fig. 2:**
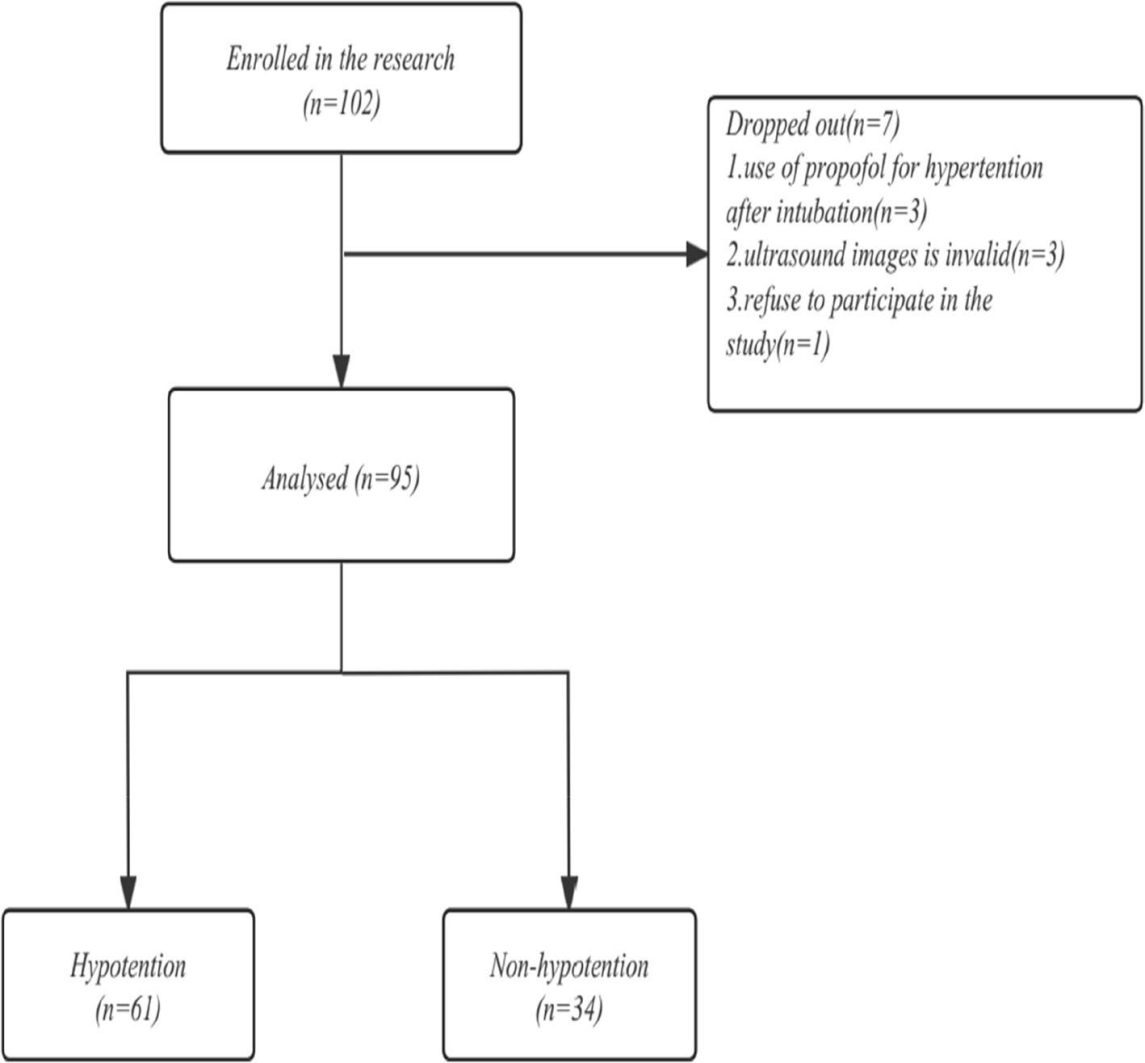
Study flow chart

**Fig. 3:**
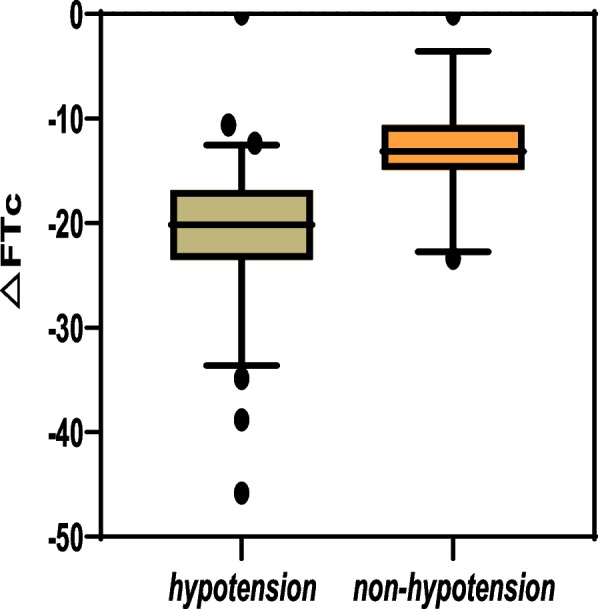
Changes in corrected carotid flow time induced by sEIOT in hypotension and non-hypotension. ΔFTc, changes in corrected carotid flow time by sEIOT

A total of 102 participants were recruited for this trial. Seven were excluded: 3 received intravenous propofol due to postintubation hypertension, 3 had inadequate ultrasound images, and 1 declined study participation (Fig. 2). Among the remaining 95 enrolled participants, there were 61 patients with hypotension and 34 with non-hypotension. Patients who developed hypotension had a higher ΔFTc in the carotid artery. The decrease in ΔFTc was greater in hypotension (−21.42 ± 6.06 mmHg) than in non-hypotension (−15.68 ± 3.59 mmHg) (*p* < 0.001) (Fig. 3).

As shown in Table 1, there was no significant differences in age, height, weight, BMI, sex, ASA physical status, hypertension, diabetes, smoking history, baseline SBP, DBP, baseline MAR, and baseline HR between the hypotension and the non-hypotension groups (*P* > 0.05).

**Table 1:** Baseline demographic and hemodynamic data between the hypotension and non-hypotension groups

Parameter	All patients (*n* = 95)	Hypotension (*n* = 64)	Non-hypotension (*n* = 31)	*P* value
Age (years)	56.66 ± 7.44	57.42 ± 6.18	55.20 ± 9.32	0.15
BMI (kg/m2)	23.87 ± 3.04	24.22 ± 3.34	23.99 ± 3.13	0.59
Male/female	39/63	27/40	12/23	0.55
ASA physical status, II/III	80/22	51/16	29/6	0.43
Hypertension	30/72	23/44	7/28	0.13
Diabetes	17/85	10/57	7/28	0.51
Smoker(yes/no)	16/86	10/57	6/29	0.77
Baseline SBP (mmHg)	142.52 ± 20.73	142.93 ± 21.76	141.74 ± 18.89	0.79
Baseline DBP (mmHg)	82.54 ± 11.20	80.37 ± 11.22	82.54 ± 11.20	0.36
Baseline MAP (mmHg)	101.07 ± 13.38	100.59 ± 13.62	101.97 ± 13.06	0.62
Baseline HR (bpm)	76.05 ± 12.34	76.03 ± 12.60	77.40 ± 11.96	0.60

*BMI* Body Mass Index, *ASA* American Society of Anesthesiologists, *HR* Heart rate, *SBP* Systolic blood pressure, *DBP* Diastolic blood pressure, *MAP* Mean arterial pressure

**Fig. 4:**
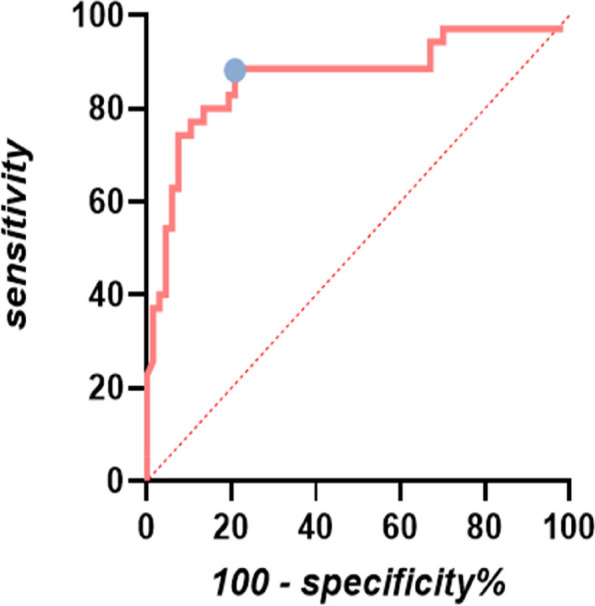
Receiver operating characteristic curves showing the predictability of carotid artery FTc on hypotension after induction of anesthesia in patients. The circle on the curve indicates the optimal cutoff values determined by maximizing the Youden index. AUC, area under the receiver operating characteristic curve

**Fig. 5:**
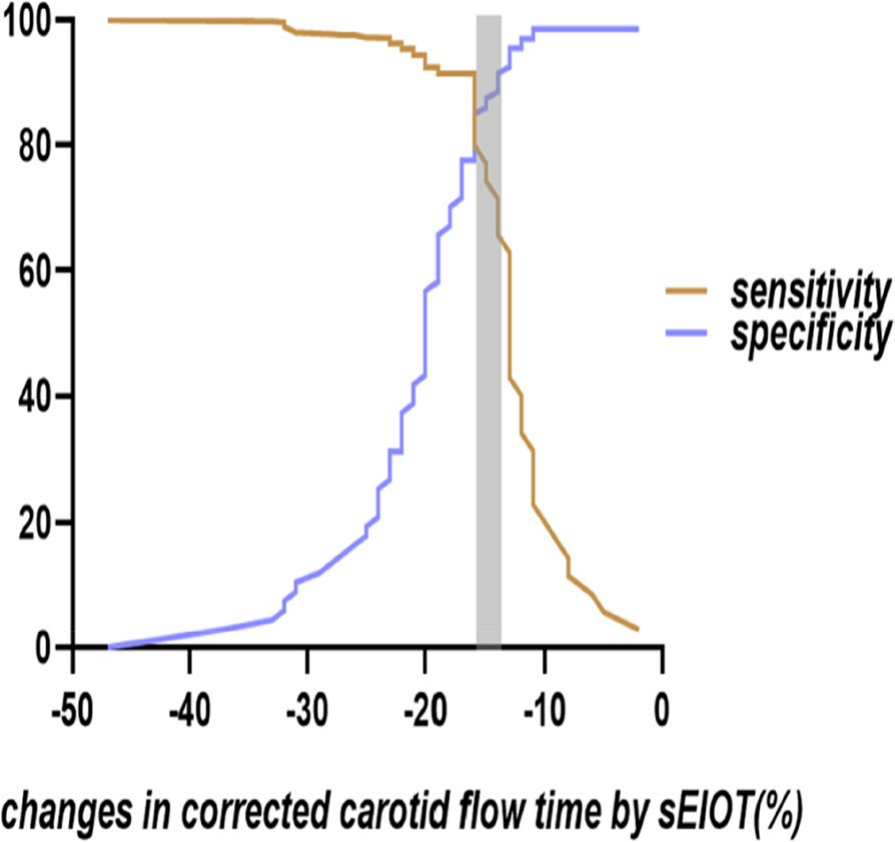
Gray zone for ΔFTc. The purple and brown lines indicate specificity and sensitivity, respectively. The gray zone indicates the inconclusive range of measurement values. ΔFTc, changes in corrected carotid flow time by sEIOT

The ability of corrected carotid flow time change (ΔFTc) to predict PIH is illustrated in Fig. 4. The AUC for carotid artery ΔFTc was 0.88 (95%CI, 0.81 to 0.96; *P* < 0.001), and the optimal cutoff value was -16.57%, with a sensitivity of 91.40% and specificity of 77.60%. The x-axis represents the percentage change in carotid corrected blood flow time, while the y-axis represents sensitivity and specificity for analysis purposes depicted in Fig. 5. A gray zone was shaded to indicate ΔFTc values where either sensitivity or specificity fell below 90%. This gray region ranged from -16.34% to -15.36% and encompassed approximately 14% of patients, including 13 identified explicitly within this range group based on their characteristics. Regarding ΔCDPV, its predictive capability for PIH demonstrated a modest AUC level at only 0.54, with an optimal cutoff value set at -1.47%. Sensitivity and specificity values were relatively low at approximately 55.20% and 57.10% respectively, when using ΔCDPV to predict PIH.

**Fig. 6:**
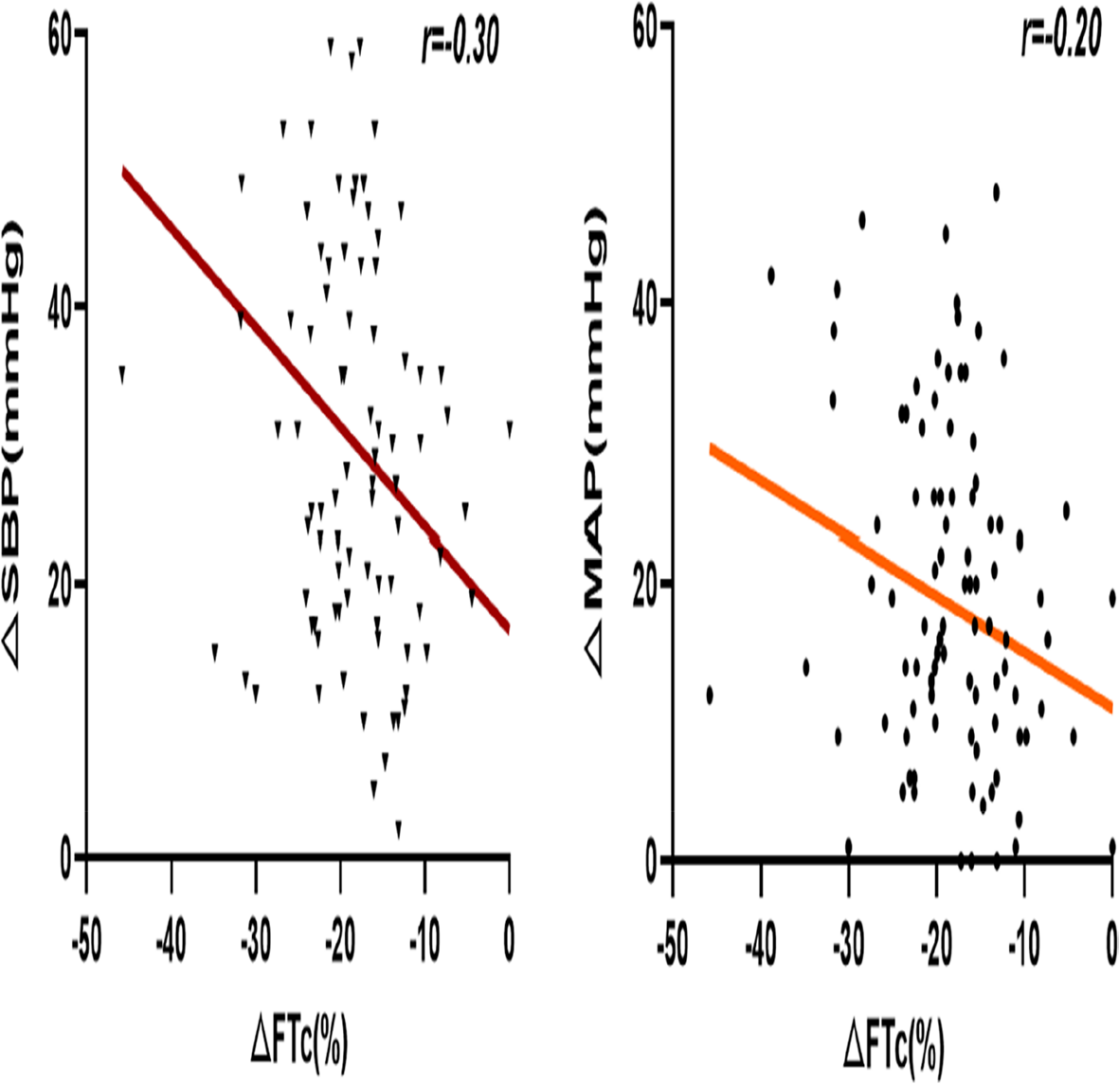
Scatter plots demonstrating the associations between ΔSBP and ΔMAP with decreases in ΔFTc relative to baseline following general anesthesia induction

The percentage change in carotid corrected blood flow time was plotted on the x-axis, while the changes in SBP and MAP from baseline at 3 min after intubation were plotted on the y-axis to create a scatter trend plot (Fig. 6). Pearson correlation analysis was employed to examine the relationship between ΔFTc and ΔSBP, ΔDBP, and ΔMAP at 3 min after intubation. The results revealed a negative correlation between ΔFTc and both ΔSBP and ΔMAP, but no significant correlation with ΔDBP. The correlation coefficient for ΔFTc and ΔSBP was *r* = -0.30 (*p* = 0.02), with a linear regression equation of Y = 16.73–0.73X. Similarly, the correlation coefficient for ΔFTc and ΔMAP was *r* = -0.20 (*p* = 0.04), with a linear regression equation of Y = 11.10–0.40X. Due to some patients experiencing postintubation hypertension at 3 min after intubation, vasoactive drugs or rehydration were promptly administered, which may have influenced subsequent blood pressure values; therefore, only the correlation analysis between ΔFTc and ΔSBP, ΔDBP, and ΔMAP at 3 min was conducted.

As indicated in Table 2, the multivariate logistic regression analysis results showed no statistically significant differences in BMI, fasting time, baseline DBP MAP, and ΔCDPV (*P* > 0.05). However, baseline SBP and baseline FTc was independent predictor of hypotension after the induction of general anesthesia, with odds ratios of 1.12 (95% CI: 1.03–1.21; *P* < 0 0.001) and 0 0.91(95%CI: 0.86 -0 0.94; *P* < 0.001), respectively.

**Table 2:** Results of logistic regression analysis to predict the occurrence of hypotension after induction of anesthesia

Parameter	OR	95%CI	*P* value
Age	1.03	0.97–1.09	0.34
BMI	0.99	0.86–1.15	0.98
Fasting time	0.98	0.88–1.10	0.73
Baseline SBP	1.12	1.03–1.21	< 0.001**
Baseline DBP	0.99	0.93–1.05	0.65
Baseline MAP	0.97	0.90–1.05	0.50
Baseline FTc	0.91	0.86–0.94	< 0.001**

*OR* Indicates odds ratio, *CI* Confidence interval, *BMI* Body Mass Index, *SBP* Systolic blood pressure, *DBP* Diastolic blood pressure, *MAP* Mean arterial pressure, *FTc* Corrected flow time

**P < 0.01

## Discussion

This study demonstrated that carotid ΔFTc following a simulated end-inspiratory occlusion maneuver assessed by Doppler ultrasound was a valid predictor for predicting hypotension after anesthetic induction in ASA II-III patients for gastrointestinal tumor surgery. However, the ΔCDPV was a poor predictor of postinduction hypotension. The optimal cutoff value of ΔFTc was -16.57%, with a sensitivity of 91.4% and specificity of 77.60%. The gray zone for carotid artery ΔFTc was -16.34% to -15.36% and included 14% of the patients.

In our study, PIH was defined as a decrease in SBP and MAP exceeding 30% of the baseline value or MAP < 60 mmHg from the initiation of anesthesia to 10 min after intubation. This criterion has been shown to be consistent with an increase in postoperative adverse events. Since hypovolemia is an important cause of PIH, all patients adopt a consistent intravenous fluid infusion protocol before induction to reduce the impact of fluid infusion on PIH [13]. The study enrolled patients undergoing elective gastrointestinal tumor surgery who needed bowel preparation and prolonged fasting due to the risk of gastrointestinal bleeding, which could potentially result in insufficient baseline circulatory volume. Inadequate management may lead to hemodynamic instability and an increased likelihood of complications. We hypothesize that preoperative hypovolemia may contribute to the development of PIH, particularly in gastrointestinal tumor patients with inadequate baseline volume. Propofol, chosen as the anesthesia induction agent due to its widespread clinical use and known ability to induce hypotension by directly dilating veins and inhibiting sympathetic nerves, is expected to significantly affect patients with hypovolemia [18].

The volume assessment presents a challenge for clinician. Conventional static parameters such as central venous pressure is invasive or susceptible to inaccuracies. Ultrasonic carotid blood flow indicators have become popularity in guiding clinical practice in recent years. Carotid artery FTc and ΔCDPV accurately assess volume response and exhibit a high predictive value for PIH [25]. Unlike other parameters that require specialized equipment and are minimally invasive, ultrasound common carotid FTc is a relatively new noninvasive and rapid predictor of fluid responsiveness, applicable even to spontaneously breathing patients [1, 14]. A seminal study by Blehar et al. [26] demonstrated an increase in carotid FTc with fluid resuscitation among dehydrated patients. The Pace R et al. [27] experiments also demonstrated that the changes in ΔVTI an ΔDPV of the aorta and carotid artery can be reliable predictors of fluid responsiveness in mechanically ventilated patients with unstable blood flow. Additionally, the trial conducted by Wang et al. [24] revealed that changes in corrected carotid blood flow time and peak blood flow velocity during respiration can predict hypotension after the induction of general anesthesia in elderly patients. Recently, corrected carotid flow time changes have been recognized as a reliable indicator of fluid responsiveness. Therefore, intervention targeting on FTc alterations may have predictive value for PIH.

Several functional hemodynamic tests have emerged to assess fluid responsiveness by manipulating cardiac preload. Some examples include the passive leg raising test and various maneuvers that alter intrathoracic pressure, such as transient increases in tidal volume, recruitment maneuvers, sighing maneuvers, or end-expiratory obstruction test, all of which can induce changes in cardiac preload [21, 22, 28–30]. End-expiratory occlusion (EEO) and end-inspiratory occlusion (EIO) tests have been effectively utilized for predicting fluid responsiveness across different clinical settings using calibrated pulse curve analysis and echocardiography [31]. The EIO test is a conventional method based on the principle of heart-lung interaction during spontaneous breathing and mechanical ventilation. A study by François Dépret [32] found that a cumulative percentage change greater than 9% in cardiac index caused by two consecutive end-inspiratory and end-expiratory block maneuvers could indicate patients fluid responsiveness. Our study showed that the change in FTc induced by sEIOT was a reliable predictor of hypotension following general anesthesia induction. Aya et al. [21] demonstrated that changes in carotid artery blood flow time induced by lung recruitment maneuvers could be utilized to assess fluid responsiveness in patients, which is consistent with the findings of our study. Furthermore, Aya’s research revealed that the area under the ROC curve of ΔFTc was 0.82, exhibiting an optimal cutoff value of -11.7%, which close to the observed area under the curve and optimal cutoff value in this study. However, it is essential to note that our trial identified a higher optimal cutoff value for ΔFTc compared to the findings reported by Aya et al., potentially due to the spontaneously breathing patients during sEIOT in our study, and the spontaneous breathing may affect ΔFTc.

Individual variations resulting from different maximum inspiratory volumes can significantly impact intrathoracic pressure fluctuations, influencing venous blood return to the heart and leading to substantial variation in stroke volume, thereby affecting ΔFTc measurements. In current clinical practice, empirical treatment measures are predominantly utilized for managing postinduction hypotension. This study establishes a preoperative prediction model for PIH by integrating preoperative ultrasound measurements of carotid artery parameters with a simple inspiratory occlusion test. It provides valuable ways to the prevent and mitigate the adverse effects associated with PIH.

## Limitations

Firstly, hemodynamic monitoring uses noninvasive blood pressure, which may be biased. Secondly, long-term hypertensive patient with mild to moderate hypertension may increase left ventricular afterload. Additionally, a potential inverse correlation between FTc and systemic vascular resistance could have implications for FTc [18, 33]. Finally, this study was conducted in adult patients with preserved cardiopulmonary function, and further investigation is warranted to ascertain its prognostic value in geriatric patients, those afflicted with severe cardiovascular pathologies, and patients experiencing hemodynamic instability [34]. In addition, applying dynamic monitoring in clinical practice and optimizing guided fluid therapy requires further investigation.

## Conclusion

In conclusion, ΔFTc induced by sEIOT is a dependable predictor of hypotension following the induction of general anesthesia in patients undergoing elective gastrointestinal tumor resection, and ΔCDPV has less predictive value. Future investigations should explore its applicability in high-risk patients through integrated fluid therapy strategies and dynamic monitoring.

## Data Availability

The datasets used and/or analyzed in the current study are available from the corresponding author on reasonable request.

## Abbreviations

sEIOT: Simulated end-inspiratory occlusion test
PIH: Post-induction hypotension
FTc: Corrected flow time
CDPV: Peak blood flow velocity
BP: Blood pressure
HR: Heart rate
SBP: Systolic blood pressure
DBP: Diastolic blood pressure
MAP: Mean arterial pressure
SVV: Stroke volume variation
ROC: Receiver operating characteristic
AUC: Area under the receiver operating characteristic curve
ASA: American Society of Anesthesiologists
IVC-CI: Inferior vena cava collapse index
VTI: Velocity Time Integral
sEEOT: Simulated end-expiratory occlusion test
FT: Flow time
BMI: Body Mass Index
PCV-VG: Pressure controlled volume guaranteed ventilation
VT: Tidal volume
RR: Respiratory rate

## References

[1] Singla D, Gupta B, Varshney P (2023). Role of carotid corrected flow time and peak velocity variation in predicting fluid responsiveness: a systematic review and meta-analysis. Korean J Anesthesiol.

[2] Park JY, Yu J, Kim CS (2023). Effect of pneumatic leg compression on post-induction hypotension in elderly patients undergoing robot-assisted laparoscopic prostatectomy: a double-blind randomised controlled trial. Anaesthesia.

[3] Okamura K, Nomura T, Mizuno Y (2019). Pre-anesthetic ultrasonographic assessment of the internal jugular vein for prediction of hypotension during the induction of general anesthesia. J Anesth.

[4] Padley JR, Ben-Menachem E (2018). Low pre-operative heart rate variability and complexity are associated with hypotension after anesthesia induction in major abdominal surgery. J Clin Monit Comput.

[5] Kaydu A, Güven DD, Gökcek E (2019). Can ultrasonographic measurement of carotid intima-media thickness predict hypotension after induction of general anesthesia?. J Clin Monit Comput.

[6] Lee J, Woo J, Kang AR (2020). Comparative analysis on machine learning and deep learning to predict post-induction hypotension. Sensors (Basel).

[7] Gibson LE, Mitchell JE, Bittner EA (2023). An assessment of carotid flow time using a portable handheld ultrasound device: the ideal tool for guiding intraoperative fluid management?. Micromachines (Basel).

[8] Südfeld S, Brechnitz S, Wagner JY (2017). Post-induction hypotension and early intraoperative hypotension associated with general anaesthesia. Br J Anaesth.

[9] Li XF, Huang YZ, Tang JY (2021). Development of a random forest model for hypotension prediction after anesthesia induction for cardiac surgery. World J Clin Cases.

[10] Erlich C, Lamer A, Moussa MD (2022). End-tidal carbon dioxide for diagnosing anaphylaxis in patients with severe postinduction hypotension. Anesthesiology.

[11] Miyazaki R, Sumie M, Kandabashi T (2019). Resting pupil size is a predictor of hypotension after induction of general anesthesia. J Anesth.

[12] Shao L, Zhou Y, Yue Z (2022). Pupil maximum constriction velocity predicts post-induction hypotension in patients with lower ASA status: a prospective observational study. BMC Anesthesiol.

[13] Zhang J, Critchley LA (2016). Inferior vena cava ultrasonography before general anesthesia can predict hypotension after induction. Anesthesiology.

[14] Beier L, Davis J, Esener D (2020). Carotid ultrasound to predict fluid responsiveness: a systematic review. J Ultrasound Med.

[15] Schleifer JI, Selame LAJ, Short Apellaniz J (2021). Sonographic assessment of the effects of mechanical ventilation on carotid flow time and volume. Cureus.

[16] Jung S, Kim J, Na S (2021). Ability of carotid corrected flow time to predict fluid responsiveness in patients mechanically ventilated using low tidal volume after surgery. J Clin Med.

[17] Sidor M, Premachandra L, Hanna B (2020). Carotid flow as a surrogate for cardiac output measurement in hemodynamically stable participants. J Intensive Care Med.

[18] Maitra S, Baidya DK, Anand RK (2020). Carotid artery corrected flow time and respiratory variations of peak blood flow velocity for prediction of hypotension after induction of general anesthesia in adult patients undergoing elective surgery: a prospective observational study. J Ultrasound Med.

[19] Mackenzie DC, Khan NA, Blehar D (2015). Carotid flow time changes with volume status in acute blood loss. Ann Emerg Med.

[20] Kenny JS, Barjaktarevic I, Eibl AM (2020). A wearable carotid Doppler tracks changes in the descending aorta and stroke volume induced by end-inspiratory and end-expiratory occlusion: a pilot study. Health Sci Rep.

[21] Kimura A, Suehiro K, Juri T (2022). Changes in corrected carotid flow time induced by recruitment maneuver predict fluid responsiveness in patients undergoing general anesthesia. J Clin Monit Comput.

[22] D’Arrigo S, Dell’Anna AM, Sandroni C (2023). Can carotid artery Doppler variations induced by the end-expiratory occlusion maneuver predict fluid responsiveness in septic shock patients?. Crit Care.

[23] Mehandale SG, Rajasekhar P (2017). Perfusion index as a predictor of hypotension following propofol induction - a prospective observational study. Indian J Anaesth.

[24] Wang J, Li Y, Su H (2022). Carotid artery corrected flow time and respiratory variations of peak blood flow velocity for prediction of hypotension after induction of general anesthesia in elderly patients. BMC Geriatr.

[25] Shen J, Dai S, Tao X (2022). Corrected flow time and respirophasic variation in blood flow peak velocity of radial artery predict fluid responsiveness in gynecological surgical patients with mechanical ventilation. BMC Anesthesiol.

[26] Blehar DJ, Glazier S, Gaspari RJ (2014). Correlation of corrected flow time in the carotid artery with changes in intravascular volume status. J Crit Care.

[27] Pace R, Lassola S, Miori S (2022). Carotid vs. aortic velocity time integral and peak velocity to predict fluid responsiveness in mechanically ventilated patients. A comparative study. Minerva Anestesiol.

[28] Antiperovitch P, Iliescu E, Chan B (2017). Carotid systolic flow time with passive leg raise correlates with fluid status changes in patients undergoing dialysis. J Crit Care.

[29] Messina A, Colombo D, Barra FL (2019). Sigh maneuver to enhance assessment of fluid responsiveness during pressure support ventilation. Crit Care.

[30] Messina A, Montagnini C, Cammarota G (2019). Tidal volume challenge to predict fluid responsiveness in the operating room: an observational study. Eur J Anaesthesiol.

[31] Horejsek J, Balík M, Kunstýř J (2023). Prediction of fluid responsiveness using combined end-expiratory and end-inspiratory occlusion tests in cardiac surgical patients. J Clin Med.

[32] Dépret F, Jozwiak M, Teboul JL (2019). Esophageal Doppler can predict fluid responsiveness through end-expiratory and end-inspiratory occlusion tests. Crit Care Med.

[33] Kim DH, Shin S, Kim N (2018). Carotid ultrasound measurements for assessing fluid responsiveness in spontaneously breathing patients: corrected flow time and respirophasic variation in blood flow peak velocity. Br J Anaesth.

[34] Kim HJ, Cho AR, Lee H (2022). Ultrasonographic carotid artery flow measurements as predictors of spinal anesthesia-induced hypotension in elderly patients: a prospective observational study. Med Sci Monit.

